# Management of COVID-19-related challenges faced by EMS personnel: a qualitative study

**DOI:** 10.1186/s12873-021-00489-1

**Published:** 2021-08-14

**Authors:** Fateme Mohammadi, Banafsheh Tehranineshat, Mostafa Bijani, Ali Asghar Khaleghi

**Affiliations:** 1 Department of Nursing, Hamadan University of Medical Sciences, Chronic Diseases (Home Care) Research Center and Autism Spectrum Disorders Research Center, Hamadan, Iran; 2 Community-based Psychiatric Care Research Center, Department of Nursing, School of Nursing and Midwifery, Shiraz University of Medical Sciences, Shiraz, Iran; 3 Department of Medical Surgical Nursing, Fasa University of Medical Sciences, Fasa, Iran; 4 Non Communicable Diseases Research Center (NCDRC), Fasa University of Medical Sciences, Fasa, Iran

**Keywords:** COVID-19, Emergency medical services, Qualitative research

## Abstract

**Background:**

As the first link in the chain of providing healthcare services in the frontline of the battle against COVID-19, emergency medical services (EMS) personnel are faced with various challenges, which affect their professional performance. The present study aimed to identify some strategies to manage the COVID-19-related challenges faced by the pre-hospital emergency care personnel in the south of Iran.

**Methods:**

In this qualitative descriptive study, 27 pre-hospital emergency care personnel who were selected through the purposeful sampling method. Data were collected through 27 semi-structured, in-depth, individual interviews. The collected data were then analyzed based on the Granheim and Lundman’s method.

**Results:**

Analysis of the data resulted in the identification of 3 main themes and eight sub-themes. These three main themes were as follows: comprehensive and systematic planning, provision of medical equipment, and reduction of professional challenges.

**Conclusion:**

The findings of the present study showed that, during the COVID-19 crisis, emergency medical services personnel should be provided with a comprehensive and systematic protocol to provide pre-hospital care and their performance should be assessed in terms of a set of scientific standards. Due to lack of equipment and work overload in the current crisis, emergency medical services personnel are faced with many psychological challenges, which adversely affect their quality of pre-hospital emergency care. Furthermore, emergency care senior managers should develop comprehensive protocols, provide more equipment, and eliminate professional challenges to pave the ground for improving the quality and safety of the healthcare services in pre-hospital emergency care during the current COVID-19 crisis.

**Supplementary Information:**

The online version contains supplementary material available at 10.1186/s12873-021-00489-1.

## Background

The term “emerging” is used with various definitions to describe an infectious disease which appears for the first time in the world, one area or one population, an also for existing infectious disease, which has recently become more severe, or a pathogenic infection whose geographic range is expanding [1]. By starting 2019 from China, the emerging disease of COVID-19 has quickly spread in many parts of the world. World Health Organization (WHO) has called it a pandemic with the proportions of a Public Health Emergency of International Concern [2]. Of note, emergency medical services (EMS) personnel play a key part in the management of public health crises, including the outbreak of infectious and contagious diseases [3]. They are known as the first link in the chain of healthcare services and are currently in the frontline of providing care for patients infected with the highly infectious coronavirus, and are, therefore, exposed to significant psychological tension and many challenges in the management of COVID-19, which consequently affect their performance [4]. Iran outbreak of COVID-19 disease is complex, Iran was the third country with the highest number of reported COVID-19 cases after China and Italy at the beginning of the pandemics, whereas it has been heavily hit by the virus and now facing the fifth wave of the COVID-19 outbreak compared with other countries that are currently facing the second wave. So that is about 3.577.000 people in Iran that were infected by the virus and over 87.600 patients died [5].

A review of literature showed that not many studies with a qualitative approach have addressed the challenges faced by EMS personnel during the COVID-19 crisis. Qualitative studies can help in achieving an in-depth understanding on a concept and determining its various aspects as this type of research sets out to explore a concept in its cultural context from the perspective of those individuals who have had prolonged engagement with that concept in question and have in-depth experiences of it [6]. Thus, a qualitative approach can be employed to discover the challenges of coping with COVID-19 for EMS personnel, which subsequently results in a better understanding on their issues and concerns.

It will not be possible to improve the quality of pre-hospital emergency care without having an understanding on the rich experiences of emergency care personnel regarding the clinical challenges of the management of the COVID-19 crisis. Understanding the feelings and concerns of EMS personnel in a health crisis following the outbreak of an infectious or contagious disease like the current COVID-19, can help in developing effective strategies to manage the crisis and improve the quality of pre-hospital emergency care. Accordingly, the present study used a qualitative approach for an in-depth description of the challenges faced by EMS personnel during coping with the COVID-19 crisis in the south of Iran.

## Methods

The present study uses a descriptive qualitative design which is an effective method to obtain insight into a research question and establish the subjects’ perception of who, what, place of events, or experiences [7]. When little is known about informants’ perception of a phenomenon, a descriptive qualitative design can help researchers relate to human experiences in their unique context [8].

### Participants

In this study, 27 pre-hospital emergency care personnel who had been selected via purposeful sampling were interviewed from May 2020 to November 2020. The inclusion criteria were having at least 1 year’s experience of practice in emergency care services and willingness to participate in this study. In Iran, the education of emergency medical services’ persons is represented by associate’s or bachelor’s degree in emergency medicine, bachelor’s degree in nursing, anesthesia or as an operating room nurse. They have a certificate of professional competence to work in the pre-hospital setting. They also should not have any physical disabilities or mental disorders.

The interviews were conducted in Persian language. For translation the forward–backward method was used. Finally, the authors (the first two translators and a person from the emergency medical services) compared the interviews in English and Persian languages and agreed on their conceptual similarity.

### Data collection

Data were collected using 27 individual, semi-structured interviews. The interviews were performed by WhatsApp mobile software. The interviews were made by one of the researchers (FM). Each interview began with four general questions, including “Can you describe one of your work days in pre-hospital emergency care during the COVID-19?”, “what challenges are EMS personnel faced during the COVID-19 crisis?”, “What are your feelings when you are providing care to a patient with COVID-19?” and “What problems may arise when you are providing care to a patient infected with the coronavirus? Then, based on the answers to these questions, to clarify the information several follow-up questions were asked such as: “Can you elaborate on that?” “What do you mean?” “Can you give me an example?” In this study, the longest and shortest interviews were 45 and 30 min, respectively. (Additional file 1: Interview Guide and Question).

### Ethical considerations

The present study was conducted in terms of the principles of the revised Declaration of Helsinki, a statement of ethical principles that directs physicians and other participants in medical research involving human subjects. Prior to performing the interviews, all the subjects were informed about the objectives of the study, the voluntary nature of their participation, the data collection methods, the reason of recording the interviews, the role of the interviewer and the participants, and confidentiality and anonymity of the information. Subsequently, they were asked to sign the informed consent form if they were willing to participate. The participants were notified that they are free to withdraw from the study at any time.

### Data analysis

The present study used the conventional content analysis method suggested by Granheim and Lundman (2004) [9]. Qualitative content analysis is a research method for conceptual interpretation of the content of textual data through systematic classification, coding and theme development or designing known models toward obtaining rich, in-depth information about the phenomenon under study [10]. Accordingly immediately after each interview, the first author (FM) transcribed the recorded content. At the reading stage, the transcripts were carefully read line by line and the significant paragraphs were marked to stand out. The words, sentences, or paragraphs that carried significance regarding the challenges of coping with COVID-19 in pre-hospital emergency care were also selected as meaning units. Each meaning unit was assigned a code. At the next stage, the second author (BT) reviewed the transcripts and verified the unit meanings and codes. The codes that were found to be similar and homogenous were then merged to form categories. To confirm the reliability of the codes, the researchers reviewed the categories and compared them with the primary data. Eventually, in several joint meetings, after contemplating and comparing the categories, the research team extracted the obtained themes. The collected data were finally analyzed in MAXQDA v. 2007. (Table 1, Example of data analysis process).

**Table 1:** An example of coding and development of categories and themes

Meaning units	Coding	Category	Theme
Sometimes, we do not even have a pair of gloves, a simple mask or a special gown and go on missions in great fear. The severe shortage of PPE in pre-hospital emergency care prevents the personnel from providing quality care to COVID-19 patients (Participant 21)	Shortage of personal protective equipment	Provision and distribution of adequate PPE for the personnel	Provision of medical equipment

### Rigor

The accuracy and trustworthiness of the gained data were tested using the criteria suggested by Guba and Lincoln (1985). To ensure the study’s credibility, the researchers applied prolonged engagement, member checking, and peer review. For member-checking, four pre-hospital emergency care personnel were presented with a copy of the encoded interviews, which was previously confirmed. To perform peer-checking, five experts analyzed and observed the process of data analysis who finally validated the codes and categories. To ensure dependability and confirmability, the researchers used audit trail, including precise interview techniques, accurate transcription, and peer review. Moreover, transferability was ensured by thorough and exact descriptions of the concept in question, the characteristics of the participants, the method of data collection, the method of data analysis, and the written examples of the participants’ quotes [11].

## Results

The means of the participants’ ages and lengths of work experience were 34.33 ± 6.22 years old and 9.18 ± 5.12 years, respectively. The other demographic characteristics of the participants are shown in (Table 2). Analyses of the data obtained from the interviews resulted in finding 3 themes and 8 sub-themes (Table 3).

**Table 2:** Individual characteristics of the participants

***Participants***	***Educational level***	***Work experience (years)***
P1	Bachelor’s degree in EMS	13
P2	Associate’s degree in EMS	7
P3	Bachelor’s degree in EMS	15
P4	Bachelor’s degree in EMS	5
P5	Associate’s degree in EMS	8
P6	Associate’s degree in EMS	7
P7	Bachelor’s degree in nursing	9
98	Associate’s degree in EMS	10
P9	Bachelor’s degree in EMS	8
P10	Master’s degree in nursing	18
P11	Bachelor’s degree in EMS	5
P12	Bachelor’s degree in EMS	15
P13	Associate’s degree in EMS	4
P14	Bachelor’s degree in EMS	2
P15	Bachelor’s degree in EMS	3
P16	Associate’s degree in EMS	14
P17	Bachelor’s degree in EMS	5
P18	Bachelor’s degree in nursing	6
P19	Associate’s degree in EMS	12
P20	Bachelor’s degree in EMS	15
P21	Bachelor’s degree in EMS	3
P22	Bachelor’s degree in EMS	5
P23	Associate’s degree in EMS	17
P24	Associate’s degree in EMS	13
P25	Bachelor’s degree in nursing	19
P26	Bachelor’s degree in EMS	6
P27	Associate’s degree in EMS	4

**Table 3:** Themes and subthemes extracted from content analysis

***Themes***	***Subthemes***
***Comprehensive and systematic planning***	• Development of clinical instructions on the provision and evaluation of care provided to COVID-19 patients • Promotion of public education on how to handle pre-hospital emergency care during the COVID-19 crisis • Development of protocols for supervising EMS personnel’s provision of care provided to COVID-19 patients at home
***Provision of medical equipment***	• Provision and distribution of adequate PPE for the personnel • Provision and distribution of adequate PPE for the patients • Allocation of separate ambulances to COVID-19 patients
***Reduction of professional challenges***	• Improving the psychological security of the personnel • Reducing occupational burnout and stress

### Theme 1: Comprehensive and systematic planning

From the participants’ point of view, there is an urgent need for proposing a comprehensive care protocol with clear instructions on how to deal with COVID-19 patients during the current crisis. The absence of such a protocol could adversely affect the performance of EMS personnel and the quality of care provided by them. The theme of comprehensive and systematic planning consists of the following subcategories: development of clinical instructions on the provision and evaluation of care provided to COVID-19 patients, promotion of public education on how to handle pre-hospital emergency care, and development of protocols for supervising EMS personnel’s provision of care provided to COVID-19 patients at home.

#### Development of clinical instructions on the provision and evaluation of care provided to COVID-19 patients

According to the participants, emergency care administrators and policy-makers should set a single comprehensive protocol to be followed by EMS personnel under the complicated conditions of the COVID-19 crisis. One of the participants stated that:

*“We are really confused and do not know what to do. A new code of practice comes in every day and we are told to follow this one and forget the previous one. There is not even a specifically designed comprehensive protocol for EMS personnel to tell them how to cope with COVID-19 patients and we just receive the protocols designed for other healthcare personnel. The work conditions and the powers in emergency care are different from other departments. Really, some of these protocols are not applicable for emergency care”* (Participant 4).

#### Promotion of public education on how to handle pre-hospital emergency care during the COVID-19 crisis

From the participants’ point of view, another challenge of coping with COVID-19 in pre-hospital emergency care is poor public education on requesting and handling pre-hospital emergency care in the current crisis. According to one of the participants:

*“Most people are not properly familiar with COVID-19 and have inadequate knowledge on how to deal with patients infected with the disease. Unfortunately, they do not even take the most basic precautions. They act like there is no outbreak at all. After they call emergency care and the personnel meet them at their homes, they do not keep any social distancing and do not even wear any mask or gloves on”* (Participant 6).

#### Development of protocols for supervising EMS personnel’s provision of care provided to COVID-19 patients at home

From the participants’ point of view, for a correct evaluation of EMS personnel’s performance in caring COVID-19 patients, there must be a standard scientific protocol to ensure high-quality care and the safety of these patients. One of the participants stated that:

*“There is no evaluation of the clinical care provided by EMS personnel to patients with COVID-19; they do not tell us if what we have done is right or wrong. When there is no protocol for evaluating the performance of the personnel during the current outbreak of the coronavirus, well, the personnel will never know their strengths and weaknesses and may just keep giving the wrong kind of care and put the lives of their patients at risk* “(Participant 11).

### Theme 2: Provision of medical equipment

In the present study, the second theme was the provision of medical equipment, which consists of the following three subcategories: provision and distribution of adequate Personal Protective Equipment (PPE) for the personnel, provision and distribution of adequate PPE for the patients, and allocation of separate ambulances to COVID-19 patients.

#### Provision and distribution of adequate PPE for the personnel

As the participants of this study stated, EMS personnel’s occupational health in the face of the coronavirus mostly depends on their access to enough PPE. According to one of the participants:

*“We are literally dicing with death under these hard conditions brought about by the current outbreak of COVID-19. Sometimes, we do not even have a pair of gloves, a simple mask or a special gown and go on missions in great fear. The severe shortage of PPE in pre-hospital emergency care prevents the personnel from providing quality care to COVID-19 patients. How do you expect the personnel to worry about someone else’s life and safety when their own lives and safety are at higher risk? Safety comes first”* (Participant 21).

#### Provision and distribution of adequate PPE for the patients

From the participants’ perspective, another major challenge affecting the quality of care provided to COVID-19 patients in pre-hospital emergency care is the inadequacy of PPE for patients. One of the participants stated that:

*“On most of our missions where we are present at the side of a patient with suspected or the confirmed COVID-19 infection, we have inadequate protective equipment for the patient. The patient usually has a violent cough and respiratory secretions, but we cannot give him/her a mask or the special gown. The patients and their companions protest and say, “Why don’t you have the necessary tools and equipment? What is this? How are you going to save us in this pandemic if you do not have enough equipment to protect the patients?”* (Participant 13).

#### Allocation of separate ambulances to COVID-19 patients

From the participants’ point of view, another key issue in the proper management of the COVID-19 crisis in pre-hospital emergency care is lack of ambulances that are especially designated for COVID-19 cases. According to one of the participants:

*“We are experiencing a really hard time giving healthcare services to COVID-19 patients. We use the same ambulances to transfer a COVID-19 patient and a patient with a heart disease. Most of our time is spent on disinfecting the ambulance, and even then, we cannot follow all the guidelines stated in the protocol for disinfecting ambulances, because we have inadequate disinfectants. Sometimes, we have to go on a mission simultaneously when we are disinfecting the ambulance”* (Participant 23).

Another participant stated that:

*“On most of our missions, the patients complain and ask why we don’t have special ambulances for COVID-19 patients. Sometimes, patients refuse to be transferred to the hospital by the emergency care personnel and say, “Do you want to use the same ambulance that is used for those who have COVID-19 to take us to the hospital? What kind of emergency services is this? You do not even have enough equipment and ambulances”* (Participant 16).

### Theme 3: Reduction of professional challenges

The final theme extracted from the data is the reduced professional issues with the following subcategories: improving the psychological security of the personnel, reducing occupational burnout, and reducing occupational stress.

#### Improving the psychological security of the personnel

From the participants’ point of view, under the difficult conditions resulted from the outbreak of the coronavirus, EMS personnel are subjected to various kinds of psychological issues, including fear, post-traumatic stress disorder (PTSD), emotional instability, and lack of resilience. Therefore, mangers should pay more attention to the psychological security of their personnel and then they should take the necessary measures to enhance it, e.g. provision of counseling and workshops. According to one of the participants:

*“under these critical conditions, the personnel are facing different emotional and psychological problems. I myself have lost my patience on several occasions and quarreled with patients or their companions. In these hard times, no one cares about the emotional issues of the personnel or does anything constructive in this regard. As a consequence, I had insomnia for several days, I had nightmares, and suffered from fear and anxiety, and also dreaming on patients with COVID-19. When I went to a psychologist, she told me I had PTSD because of the coronavirus crisis and my working conditions”* (Participant7).

On a similar note, another participant stated that:

*“We are really exposed to lots of stress and anxiety during this coronavirus crisis and sometimes just cannot control our feelings and emotions anymore and lose our emotional stability and easily get angry. Despite the current hard conditions, nobody does anything about our emotional problems. There is not even a counselor at work whom we can talk to about our issues. Since the beginning of the outbreak, the managers have not held a single educational workshop to keep our spirits in an up level”* (Participant 18).

#### Reducing occupational burnout and stress

Two other major issues reported by the participants in this study were occupational burnout and stress. The participants mentioned that working in pre-hospital emergency care is hard and demanding and that the current COVID-19 crisis had made it even more difficult. The stress and workload during the outbreak are overwhelming and the occupational health of the EMS personnel is at risk. The large number of missions, lack of experienced staff, inadequate support provided by the managers, and poor work motivation aggravate occupational burnout and stress, which adversely affect the quality of emergency care services provided. One of the participants stated that:

*“We just do not have any energy for more works and feel drained. There is inadequate number of staff, there is inadequate equipment, and there is inadequate support. Considering all these issues, we are wiped out and there is no one helping us. Since the pandemic started, the stress level and work overload have been so high that I feel 10 years older. The mangers and authorities should do something for us. The conditions are really distressing and our occupational health is in a real danger”* (Participant 27).

According to another participant:

*“At these stressful times, the administrators have done nothing for us. They have not organized a single workshop to teach the personnel stress-management or done anything to motivate us in this situation. We have not received even a token of their appreciation”* (Participant 15).

## Discussion

The present qualitative study was conducted to identify some strategies to manage the challenges faced by EMS personnel during the outbreak of COVID-19. Analyses of the collected data showed that there is an urgent need for proposing a comprehensive care plan for managing COVID-19 patients. Moreover, emergency care administrators should develop a comprehensive evidence-based protocol for organizing and improving EMS personnel’s performance and then they should evaluate the personnel in terms of a standard protocol. Based on the experiences of the participants mentioned in the present study, provision and distribution of adequate PPE and allocation of separate ambulances for COVID-19 patients can improve the quality of pre-hospital emergency care. In addition, to reduce professional issues during the current COVID-19 crisis, healthcare administrators should take some steps to improve EMS personnel’s psychological security and control their occupational burnout. Correspondingly, these measures can improve the quality of pre-hospital emergency care and finally result in the satisfaction of patients and their families.

One of the findings of the present study was the need for a comprehensive systematic planning. From the participants’ point of view, development of a single protocol would organize and standardize EMS personnel’s practices. According to a study conducted in 2010, emergency care is not an independent body controlled by a single administrator and lacks organizational recognition and clear codes of practice, all of which are considered as structural issues in providing pre-hospital emergency care [12]. The participants of the present study believed that in order to provide the public with instructions on how to request and handle pre-hospital emergency care during the COVID-19 crisis, educational protocols should be developed. Similarly, in the study by Ventura et al. in the US (2020), most of the interviewed EMS personnel were found to be dissatisfied with the hygiene instructions noted for COVID-19 [13]. Additionally, the results of the study by Cash et al. in the US (2020) showed that, despite their education, 40% of the EMS personnel still need to be educated on N95 respirator fit testing and the use of PPE at the time of chemical, biological, and nuclear threats [14]. According to a study on EMS personnel’s attitudes toward severe infectious diseases and flu epidemics, it was reported that these healthcare professionals are not willing to work during pandemics [15, 16]. The personnel’s reluctance to work and provide care to patients with suspected infection is influenced by their beliefs on the inadequacy of PPE and other equipment and their lack of preparation to protect themselves [17, 18].

According to the participants in the present study, there must be a standard scientific protocol designed for a proper evaluation of EMS personnel’s performance. Lack of an evaluation system and failure to rank pre-hospital emergency care personnel prevent this section of healthcare system from improving. Thus, the responsible administrators should pay more attention to such factors as justice in payment, optimizing the evaluation system, improving communication and on-the-job training, promoting teamwork, and other measures, in order to empower the personnel [19].

Moreover, the establishment of an independent emergency care department and merging pre-hospital and hospital emergency services under the management and supervision of a single organization are essential to result in major improvements in the services offered by pre-hospital emergency [20] For example, just-in-time teaching for the use of PPE at the beginning of work shifts can promote continuing education in this regard [21, 22].

Another issue in pre-hospital emergency care is the shortage of equipment. The experiences of the participants in the present study showed that shortage of protective equipment during the COVID-19 crisis prevents EMS personnel from providing effective care for the infected patients. In the study by Karimi et al. (2020), lacks of support and equipment for caring COVID-19 patients were introduced as the initial concerns in clinical environments, which could also bring adverse effects on the quality of care [23]. The results of previous studies showed that inadequate PPE and medical equipment increase the incidence of infection in nurses, in this regard, a case in point is the large number of nurses and doctors who were infected and lost their lives as a result in Italy [24]. In the study by Martin-Delgado et al. (2020) conducted in three Latin American countries, nearly 70% of the included participants (doctors, nurses, and other healthcare professionals) did not have access to enough PPE, especially gowns, N95 respirators, and face shields, and 51.4% did not even have enough knowledge on how to use PPE, both of which had some adverse effects on the quality of healthcare during the pandemic [25]. According to the study by Kironji et al. (2018), ambulances lacking equipment, including burn kits, spinal boards, airways, CPR supplies, and medications are a major obstacle to provide quality pre-hospital care in low- and middle-income countries [26]. The results of the study by Eghbali et al. (2020) showed that, due to the shortage of emergency care equipment, many patients in respiratory distress do not receive proper care [27]. Therefore, Healthcare administrators should perform regular need analyses and provide the required resources according to the number of healthcare providers and the expected patients [28].

Another finding of the present study was the need for reducing professional issues. Under the current difficult conditions resulted from COVID-19, EMS personnel are suffering from different psychological issues, including depression and occupational stress. Moreover, high levels of stress and workload can put the occupational health of EMS personnel at risk and reduce the quality of care provided by them. In line with these findings, the study by Savitski et al. (2020) showed high anxiety levels in nurses [29]. Similarly, the study by Kang et al. (2020) conducted at the time of the coronavirus pandemic, reported that nurses are experiencing high levels of anxiety during the current healthcare crisis [30]. In another study (2020), nurses working in wards assigned to COVID-19 patients were found to be suffering from emotional and psychological distresses due to long working shifts, being quarantined in the hospital, not being allowed to have visitors, and practicing social distancing from their families [14].

Frontline nurses are subjected to medical hazards, including injuries, infections, and depression, which may be due to their fear of being infected, stress, and work overload associated to provide a proper care for COVID-19 patients. Accordingly, another cause of nurses’ depression can be their concern for their children and other family members [31]. In a study performed in France, a doctor had committed suicide after contracting COVID-19 [32]. The shortage of PPE increases fear of contracting the infection, which in turn, leads to some psychological disorders and occupational depression [19]. In the current health crisis frontline healthcare personnel who are directly involved in diagnosing, treating, and caring COVID-19 patients, are at higher risks of psychological traumas and disorders [33]. Among the factors that can contribute to the psychological tension of these professionals, are the daily increased number of suspected and confirmed cases of COVID-19, the increased workload, the diminished PPE supplies, extensive coverage by media, lack of specialty medication, and inadequate support received from managers [34]. In different countries, feelings of fear and anger are reported as the common psychological issues, which affect the healthcare personnel responsible for COVID-19 patients [35, 36]. As a matter of course, nurses who are in the frontline should learn personal protection methods before providing quality care [14]. There is an urgent need for an organized crisis management to minimize the psychological harms, to which the healthcare personnel are exposed during the COVID-19 crisis [37]. Therefore, healthcare policy-makers are recommended to develop more educational programs to prepare the personnel for the sudden and overwhelming requirements of caring COVID-19 patients.

### Strengths of the study

The present study was the first qualitative work of research in Iran that aimed to identify management strategies for coping with the COVID-19 crisis in pre-hospital emergency care. A deeper insight into the challenges faced by EMS personnel can help in improving the quality of pre-hospital emergency care at the time of the COVID-19 pandemic.

### Limitations

One of the limitations of the present study was that the data were collected exclusively through individual interviews with the EMS personnel of one pre-hospital emergency department in the south of Iran. In view of the economic, cultural, and social differences between Iran and other countries, it is recommended that similar studies should be conducted in other countries. It is also recommended that, in order to obtain richer data, future studies survey doctors and other members of pre-hospital emergency care, patients, and their families in other places using more varied methods of data collection, e.g. focus group interviews.

## Conclusion

The findings of the present study showed that during the COVID-19 crisis, EMS personnel should be provided with a comprehensive systematic protocol for providing pre-hospital emergency care and have their performance evaluated in terms of a standard scientific protocol designed in this regard. Due to lack of equipment and work overload, EMS personnel are subjected to many psychological issues, which adversely affect the quality of pre-hospital emergency care provided by them in the current health crisis. One of the priorities of the administrators of medical centers under these conditions should be preserving the psychological health of the personnel who are dealing with COVID-19 patients. Development of evidence-based pre-hospital care protocols and educational programs for raising the personnel’s awareness and facilitating their adaptation to the challenging conditions of the COVID-19 crisis will improve the quality of pre-hospital emergency care services.

## Supplementary Information

**Additional file 1.** Interview Guide and Question.

## Data Availability

The datasets used and/or analysed during the current study are available from the corresponding author on reasonable request.

## Abbreviations

WHO: World Health Organization
COVID-19: Coronavirus disease 2019
EMS: Emergency medical services
PPE: Personal protective equipment
PTSD: Post-traumatic stress disorder
