# Supplementary material for: Management of COVID-19-related challenges faced by EMS personnel: a qualitative study
Source: BMC Emerg Med. 2021 Aug 14;21:95. doi: 10.1186/s12873-021-00489-1 (PMC8363870; doi:10.1186/s12873-021-00489-1)
Supplement: Supplementary file 1 — Additional file 1. Interview Guide and Question. [file 12873_2021_489_MOESM1_ESM.docx]

| ***Additional file1: Interview Guide and Question*** |
| --- |
| **Interview Guide**  Thank you for accepting to be interviewed by us. The study we are undertaking is to understand more about the “Management of COVID-19-Related Challenges Faced by EMS Personnel: A  Qualitative Study”. I will be asking you several questions which are relevant to the study. You may respond to these queries in any way you feel comfortable. It is perfectly fine if you do not want to respond. At any point during the interview, if you are not clear about any questions, you are free to clarify the same with us and ask us to explain further. The information obtained during the interview will be kept confidential and will be shared only with the research team. We would like to audio record the interview in order to ensure that we do not miss out any salient issues. The recordings will be kept confidential. Your identity will be protected and your interview will also be labeled in codes. Is it OK with you that we audio record the interview?  **Interview questions**:   1. “Can you describe one of your typical workdays in pre-hospital emergency care?” 2. “Based on your experiences, what challenges are EMS personnel faced during the COVID-19 crisis?” 3. “Based on your experience, can you suggest strategies to eliminate challenges are EMS personnel faced during the COVID-19 crisis?” 4. What are your feelings when you are providing care to a patient with COVID-19?” 5. “What problems may arise when you are providing care to a patient infected with the coronavirus? |
